# 2000–2025: A Quarter of a Century of Studies on Pet Ownership in the Amazon—Epidemiological Implications for Public Health

**DOI:** 10.3390/pathogens15010077

**Published:** 2026-01-10

**Authors:** Coline J. Vanderhooft, Eduardo A. Díaz, Carolina Sáenz, Victor Lizana

**Affiliations:** 1 Servicio de Análisis, Investigación, Gestión de Animales Silvestres (SAIGAS), Veterinary Faculty, Universidad Cardenal Herrera-CEU, CEU Universities, 46115 Valencia, Spain; 2 Escuela de Medicina Veterinaria, Colegio de Ciencias de la Salud, Universidad San Francisco de Quito (USFQ), Quito 170901, Ecuador; 3 Fundación GAIAS Europa de la Comunitat Valenciana (GAIAS), 46003 Valencia, Spain; 4 Hospital de Fauna Silvestre TUERI-USFQ, Universidad San Francisco de Quito (USFQ), Quito 170901, Ecuador

**Keywords:** Amazon biome, zoonotic pathogens, companion animals, deforestation, vector-borne diseases, environmental change, Indigenous communities, disease emergence

## Abstract

Anthropogenic pressures in the Amazon Basin are reshaping human–animal–environment interactions and increasing zoonotic disease risk. Within this One Health context, domestic dogs and cats are underrecognized contributors to pathogen circulation at the human–wildlife interface. We conducted a PRISMA-compliant systematic review of zoonotic pathogens reported in companion animals across Amazonian territories in nine countries, including literature published between 2000 and 2025 in four languages. Zoonotic pathogens showed a heterogeneous yet widespread distribution, with parasitic infections, particularly *Leishmania* spp., *Toxoplasma gondii*, and vector-borne protozoa, being the most frequently reported. A pronounced geographic bias was evident, with studies concentrated in Brazil and selected areas of the western Amazon, while large portions of the Basin remain understudied. Methodological limitations included reliance on cross-sectional designs and heterogeneous diagnostic approaches, often based solely on serology. These findings highlight the need to strengthen One Health-oriented governance frameworks that integrate animal health surveillance into environmental and public health policies. Priority actions include expanding surveillance to underrepresented regions, harmonizing diagnostic protocols, investing in regional laboratory capacity, and promoting community-based monitoring. Strengthened cross-sectoral and transboundary coordination is essential to reduce zoonotic risk and support evidence-based disease prevention in Amazonian ecosystems.

## 1. Introduction

The Amazon political, biogeographical and hydrographic region covers almost 8.5 million square kilometers [1], encompassing territories of Guyana, French Guiana, Ecuador, Bolivia, Colombia, Peru, Suriname, and Venezuela, with most of its area located within Brazil [2]. This biome harbors unparalleled biodiversity: for vertebrates alone, it holds the greatest species richness on Earth [3]. Preservation of this ecosystem has profound implications for human well-being and economic stability, owing to its ability to meet human needs at both local and global scales [4]. These so-called ecosystem services [5] include evident benefits, such as direct resource extraction, as well as less tangible functions such as climate regulation, carbon and nitrogen cycling (critical for nutrient production), maintenance of an intricate hydrological network, acting as a natural detoxicant essential for planetary homeostasis [6], and trophic balance, which serves as a mediator and barrier against disease outbreaks [5]. The Amazon is among the regions with the greatest potential to harbor zoonotic agents capable of generating outbreaks [7,8]. This circumstance means that, if the ecological balance is disrupted as a result of anthropogenic stressors linked to resource overexploitation—particularly due to habitat modification through deforestation—enzootic agents begin to affect a broader range of species, vectors proliferate, and their susceptibility to carrying pathogens increases [9,10,11,12].

Currently, humans occupy approximately 50% of the planet’s terrestrial surface; of this, an estimated 37% of natural areas remain under limited human influence, largely due to the presence of Indigenous peoples [13]. The Amazon is home to 47.4 million inhabitants, of whom 2.2 million are recognized as Indigenous peoples [1]. Indigenous communities of the Amazon, through their cultural traditions and subsistence practices, maintain close interaction with their environment, drawing directly on natural resources, ranging from basic needs such as food and shelter to specialized resources derived from empirical, inherited knowledge, such as medicinal practices [6,12,13]. Although Indigenous peoples worldwide are increasingly acknowledged in contemporary policy and discourse, persistent inequalities remain. In some regions, these communities face a dual burden of infectious and chronic diseases, exacerbated by structural barriers including poverty, geographic isolation, and systemic discrimination [14].

The impacts of anthropogenic transformations on the natural environment are profound, driving substantial biodiversity loss, particularly in intact territories [13]. Broadly, human activity includes three major negative impacts: (1) loss and alteration of natural habitats and their biodiversity; (2) overexploitation of resources such as through deforestation, mining, land-use change, and territorial occupation [15]; and (3) introduction of invasive species into native ecosystems [5]. Additional effects often cited include the proliferation or imbalance of pathogen populations, accumulation of environmental toxins (pollution), and climate change [5].

Although multiple studies assess the immune status of human populations, there is a widespread lack of information regarding the condition of the pets they live with. These animals can act as sources of direct infection, transmitting or extending the infective phase of the agent, or indirect infection, by serving as multipliers of the pathogen or attractants for vectors, thereby increasing the risk of transmission [16,17]. They also interact with wildlife, generating a flow of pathogen transmission or of strains typical of the wild environment [18].

The introduction of exotic and/or invasive species exerts particularly severe effects on ecosystems. An invasive alien species is one that becomes established in an ecosystem (outside its original range) and subsequently acts as an agent of change and a threat to native biological diversity [19]. In this context, domestic dogs (*Canis lupus familiaris*) and cats (*Felis catus*) pose threats in Amazonian ecosystems. Although now common, dogs only arrived in most parts of the Amazon relatively recently [20]. Historical accounts suggest that some Indigenous groups once kept domesticated foxes to assist in hunting large rodents. These animals disappeared following European contact, as larger introduced canids quickly replaced native species, aided by their greater hunting efficiency and the spread of diseases [20].

In the Amazon Basin, close and frequent contact between humans and their domestic dogs and cats constitutes an important factor in zoonotic risk, particularly in communities with limited health education and infrastructure. A recent study conducted in Rondônia reported that more than 74% of household dogs were infected with at least one endoparasite of zoonotic importance, including *Ancylostoma* spp. and *Toxocara canis*, while nearly half were infested with ectoparasites such as Rhipicephalus sanguineus and *Ctenocephalides felis*. These findings highlight both the high exposure of companion animals and the substantial potential for pathogen transmission to humans in rural Amazonian communities [21]. Consistent with these observations, the broader scientific literature recognizes dogs and cats as reservoirs and source of multiple zoonotic agents, including parasites acquired through predation on wildlife, thereby sustaining infection cycles among wildlife, domestic animals, and humans with important ecological and public health implications [22].

Companion animals such as dogs and cats in the Amazon are often not subject to consistent control or owner supervision. Their management is commonly classified into two categories: (1) free-roaming dogs and cats, which are owned and associated with a household but allowed to wander freely; and (2) stray dogs and cats, which lack ownership and live entirely in public spaces. While risk assessments traditionally emphasize pathogen transmission from animals to humans, reverse transmission from humans to animals (anthroponosis) is also possible and may have significant consequences for the health of domestic and wild species [18]. Accordingly, the introduction and maintenance of dogs and cats as companion animals in the Amazonian biome may pose risks not only of zoonotic disease transmission to humans but also to native carnivore species through pathogen spillover and competition [19].

This complex network of transmission routes and enzootic interactions has the potential to drive wildlife population declines and human disease outbreaks, particularly in fragmented landscapes where human activity increases contact between domestic and wild animals [20]. Such interactions can negatively affect native fauna and disrupt ecosystem balance [21,22]. These dynamics are especially critical in the Amazon, where exceptional biodiversity combined with expanding human–wildlife interfaces amplifies the risk of pathogen emergence and spillover. Consequently, integrating One Health approaches that jointly consider human, animal, and environmental health is essential for understanding and mitigating zoonotic risks associated with pet ownership in this region [23]. In this context, the present study compiles and synthesizes current evidence on pet-mediated zoonotic pathogens in the Amazon, emphasizing their potential role as bridges for pathogen transmission to the human populations with which they coexist [21,22,24,25,26,27,28].

## 2. Materials and Methods

### 2.1. Literature Search Protocol

**Figure 1:**
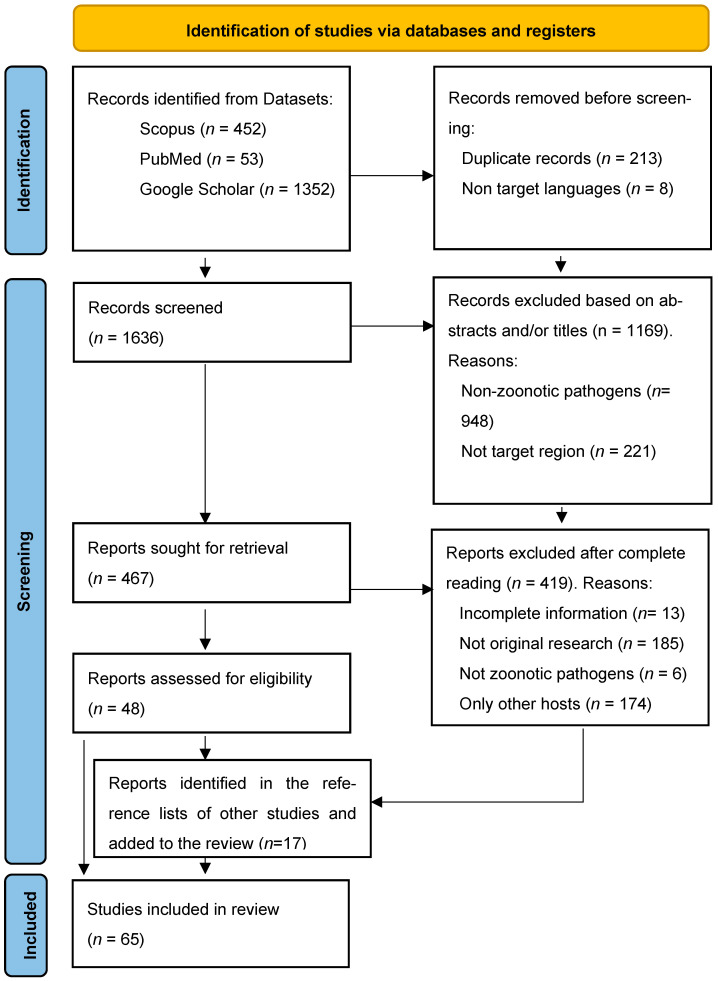
PRISMA Diagram, applying the inclusion and exclusion criteria to the results found in the selected databases. Notably, 17 results were added manually after reviewing other selected sources.

In this study, we conducted a systematic search of zoonoses related to dog and cat ownership in the Amazon over the last quarter-century, using the Preferred Reporting Items for Systematic Reviews and Meta-Analyses (PRISMA) statement protocol (Figure 1) following PRISMA 2020 guidelines. The literature search was carried out between September and October 2025 through queries in the PubMed, Google Scholar, and Scopus databases.

We systematically searched for relevant literature published in cat and dog zoonoses in this specific geographical framework. Search items were “zoonosis”, “zoonotic”, “emerging infectious disease”, “EID”, “neglected tropical disease”, “NTD”, “Amazon” and derivates (Amazonic, Amazonas, Amazonia, Amazonian), the list of the 9 countries with territories in the Amazon and finally a mention of “dog” or “canine”, “cat” or “feline”. The complete search string, including the corresponding Boolean operators, can be consulted in the Appendix A Section.

#### 2.1.1. Study Eligibility Criteria

##### Inclusion Criteria

**Figure 2:**
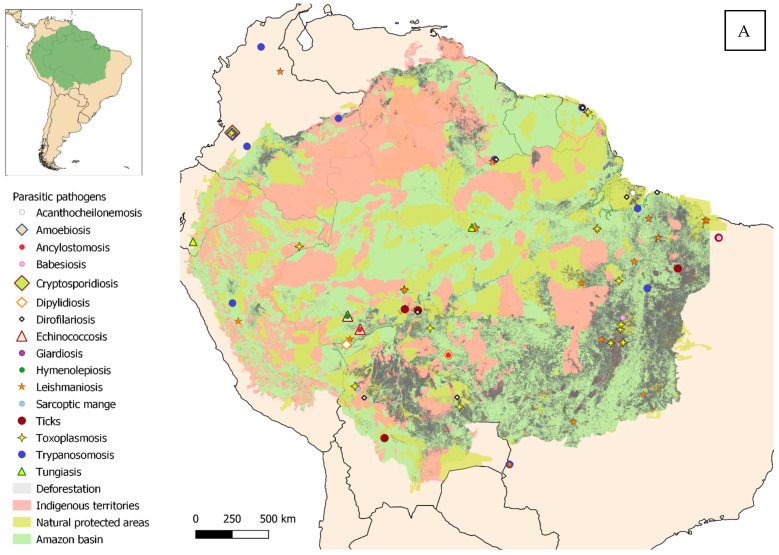

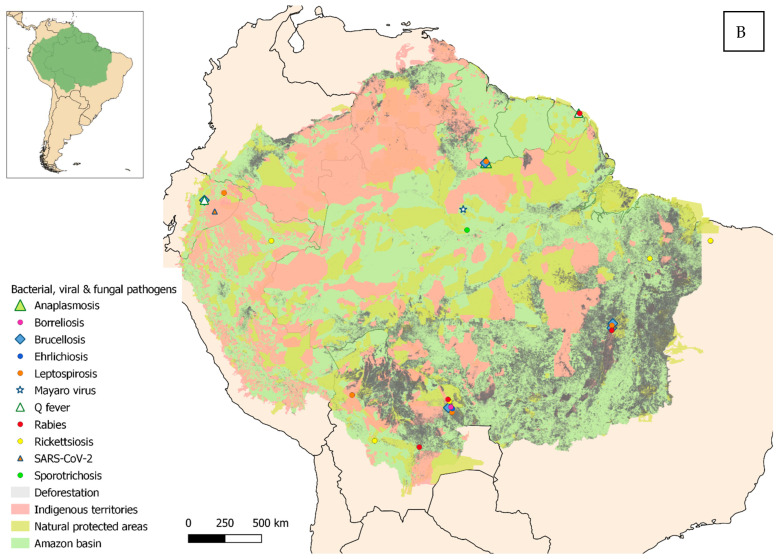
Geographical distribution of the studies reviewed, within the Amazon and surrounding areas. (**A**) Parasitic diseases. (**B**) Bacterial, viral and fungal diseases.

Eligible studies were required to address one or more zoonotic diseases and to fall within the defined geographical scope, which, according to RAISG criteria [1], corresponds to the maximum area of overlap between the Amazon biome, the hydrographic basin, and administrative boundaries (Figure 2A,B). Studies published from 2000 to October 2025, the time of manuscript preparation, and written in English, Spanish, Portuguese, or French were considered eligible. Expanding the search strategy beyond English to include other official languages of the region enabled the identification of additional relevant data and enhanced the comprehensiveness of this review.

The primary sources consisted of peer-reviewed journal articles; however, additional materials, including doctoral theses, master dissertations, and graduate monographs, were also included when they reported original data. Although such sources are often excluded from systematic reviews, their inclusion enabled the incorporation of relevant data that may not have been fully disseminated through peer-reviewed journals, thereby contributing to a more comprehensive assessment of pathogen prevalence. The methodological quality of non-peer-reviewed studies was evaluated using the same eligibility criteria applied to peer-reviewed publications, and only records reporting original data, clearly defined study populations, and extractable prevalence estimates were included. Studies with insufficient methodological detail were excluded. With exceptions of peer-reviewed case reports, studies were required to provide a clear estimate of prevalence in dogs and/or cats, even when other animal species were also investigated [1].

##### Exclusion Criteria

Records were excluded if they met any of the following criteria: duplicate entries retrieved from multiple databases (Duplicate records); articles not published in the predefined target languages (Non-target languages); studies conducted outside the predefined geographic scope (Amazon basin and peripheral area) (Non-target region); studies investigating host species other than dogs and cats (Non-target species); articles not addressing pathogens transmissible among animals and humans (Non-zoonotic pathogens); studies lacking sufficient methodological detail or extractable data to allow critical appraisal or to provide a clear estimate of pathogen prevalence (Incomplete information); or publications that did not report original primary research data (Non-original research).

Artificial intelligence-assisted tools were employed to support the identification of some exclusion criteria during screening (language); however, all records were subsequently subjected to manual verification to ensure accuracy and to identify relevant exclusions not detected automatically.

##### Data Extraction

All authors independently extracted data and discrepancies were resolved by discussion. Quantitative and qualitative data extraction from the included studies was presented as a table in an Excel spreadsheet. The extracted components encompassed the name of the pathogen/disease, country and region, prevalence, detection method, species tested (dog or cat), the primary author along with the year of publication and observations related with the relevance of the disease.

### 2.2. Review Registration

This review was not registered in any database of systematic review protocols.

### 2.3. Risk of Bias and Methodological Limitations

No formal risk of bias tool was applied due to heterogeneity of study designs; limitations are acknowledged in the Discussion.

## 3. Results

The literature search yielded a large volume of results but reading the titles and/or the abstracts allowed us to eliminate many of them in an initial phase, mainly because they were conducted outside our geographical scope, not addressing pathogens transmissible between animals and humans and/or focused on species other than those of interest. A significant number of relevant studies (19) were included manually, often as a result of reading the content of articles that referred to these investigations.

The analysis comprised 65 publications, predominantly peer-reviewed journal articles, including 52 original research studies and 7 case reports, which together accounted for 90.7% (59/65) of the included literature. In addition, six non-peer-reviewed academic works were incorporated, consisting of three doctoral dissertations, one master’s thesis, and two postgraduate monographs (9.3%; 6/65). The majority of the publications were written in English (*n* = 57), followed by Portuguese (*n* = 7) and Spanish (*n* = 1).

Most of the selected resources come from Brazil (41/65; 63.1%), followed by French Guiana (7/65; 10.7%) and Peru (6/65; 9.2%). Notably, two countries in the region (Venezuela and Suriname) are absent, contributing no research to this review. Many of the studies evaluated multiple agents simultaneously, with fungi being the least represented (only one study, 0.86%), followed by mentions of viruses (6/116; 5.1%), bacteria (25/116; 21.5%), and a clear majority of parasites (84/116; 72.4%).

Table 1, Table 2 and Table 3 summarize zoonotic viral, fungal, bacterial, and parasitic pathogens detected in domestic dogs and cats across Amazonian territories, highlighting prevalence, host species, geographic distribution, and diagnostic approaches.

Viral and fungal pathogens: Mayaro virus showed high seroprevalence in Brazil, with 60.5% of dogs and 46.1% of cats testing positive north of Manaus (ELISA-ICC). Rabies virus prevalence varied regionally: absent in French Guiana, moderate in Brazil (8–11%), and high in Bolivia (50–56%). SARS-CoV-2 was detected in Ecuadorian Amazon dogs (66.6%, RT-qPCR), indicating active circulation. *Sporothrix* spp. infections were endemic in Brazil, with 2798 confirmed feline cases, primarily in Amazonas State, demonstrating significant public health relevance.

Bacterial pathogens: Tick-borne bacteria such as *Anaplasma* spp. and *Borrelia burgdorferi* were largely absent, while sporadic *Brucella* spp. exposure was observed (2.6–10%). *Coxiella burnetii* seroprevalence reached 12.3% in French Guiana dogs. *Ehrlichia* spp. ranged from 10–14.6% in Guyana and Brazil to 86% in Bolivia. *Leptospira* spp. were widespread, particularly in Ecuador (75%), with species-specific identification of *L. borgpetersenii* serovar Hardjo and *L. interrogans*. High exposure to *Rickettsia* spp. was observed across Brazil, Bolivia, and Peru, though molecular confirmation was limited.

Parasitic pathogens. Arthropod ectoparasites (*Amblyomma* spp., *Rhipicephalus sanguineus*) were common (5–22% infestation). Helminths (*Ancylostoma*, *Toxocara*, *Trichuris vulpis*) were highly prevalent; zoonotic cestodes (*Echinococcus*, *Dipylidium*) were detected at lower prevalence. Enteric protozoa (*Cryptosporidium*, *Giardia*) were widespread in Colombia. Vector-borne protozoa, including *Babesia canis*, *Dirofilaria immitis*, *Trypanosoma cruzi*, and *T. evansi*, showed variable but sometimes very high prevalence, indicating intense enzootic circulation. *Leishmania* spp. were among the most prevalent pathogens, with multiple species across Brazil, Colombia, Peru, Guyana, and French Guiana, showing seroprevalence and molecular detection rates often exceeding 40–70% in dogs; cats were confirmed hosts in several areas. *Toxoplasma gondii* exhibited consistently high seroprevalence (60–80%) in both dogs and cats, reflecting sustained environmental contamination and transmission.

## 4. Discussion

The absence of a formal risk of bias assessment represents a limitation of this review. However, the marked heterogeneity of study designs, data sources, host species, diagnostic approaches, and geographic contexts precluded the consistent application of a single standardized risk of bias tool. The literature included in this review encompasses a wide range of evidence types, including peer-reviewed articles, case reports, and theses, which differ substantially in methodological structure and reporting standards. Applying a uniform bias assessment framework under these conditions could have resulted in misleading or non-comparable evaluations. Nevertheless, this limitation has been taken into account in the interpretation of the results, and findings are discussed with appropriate caution, emphasizing patterns and knowledge gaps rather than quantitative synthesis or causal inference.

The georeferencing of studies on a regional map graphically shows how many of the studies evaluated multiple agents simultaneously, with a clear majority of parasites. Trypanosomiasis is one of the most frequently reported diseases in our literature review. Since its discovery in 1909, it has been widely documented from the southern United States to southern Argentina [88]. It constitutes an example of an enzootic disease that, originally limited to wild animals in uninhabited areas with little human impact, has become a zoonosis following the establishment of humans and domestic animals and the associated environmental transformations [89]. Almost two hundred species of mammalian hosts, both wild and domestic, play important roles in the epidemiological cycle as reservoirs [89], with particular emphasis on opossums (*Didelphis marsupialis*, *D. albiventris*, and others), considered key hosts in numerous studies [88,90,91]. Cats and especially dogs are identified as the most relevant domestic reservoirs [92], serving as a blood source for the vectors (hematophagous triatomine bugs—*Rhodnius* spp., *Panstrongylus* spp., and *Triatoma* spp.) [16]. The presence of dogs in the household makes the habitat more attractive to vectors, which translates into a higher infection risk for the owners [17].

Together with trypanosomiasis, leishmaniosis is the most frequently reported parasitic disease in the reviewed articles, with references to a wide variety of species affecting humans and animals and showing different degrees of pathogenicity (*L. (chagasi) infantum*, *L. (V.) braziliensis*, *L. guyanensis*, *L. amazonensis*, *L. panamensis*, *L. pifanoi*) [50,59,60,71,93]. Likewise, the associated epidemiological cycles, vectors, and reservoirs can also differ—for example, the relevance of the two-toed sloth *Choloepus didactylus* or the opossum *Didelphis marsupialis* in maintaining *L. guyanensis*, vectored by *Lutzomyia umbratilis*, whereas for *L. amazonensis* the main vector is *Lutzomyia flaviscutellata* and the reservoir is the rodent *Proechimys cuvieri* [94]. In deforested areas, however, it is the dog that emerges as the preferred food source for phlebotomine sandflies, becoming the main host for the parasite [95]. In addition to autochthonous transmission, imported cases are also reported, associated with the movement of domestic dogs, as in the case of *L. infantum* in French Guiana, introduced from endemic areas in Europe [74]. Although several authors suggest a progressive increase in the presence of domestic cats in the region, the studies testing this disease remain scarce [72]. In tropical areas, the great diversity of species and strains of the genus *Leptospira* creates an extensive network of mammals involved in its maintenance and transmission [96]. Besides the well-known role of rodents (ranging from purely wild species to synanthropic ones) [97,98], other mammals have become increasingly relevant due to their close contact with humans. This is evidenced by the prevalence found in swine, cattle or equine livestock [96,99,100]. Regarding pets, given their close contact with human households, they deserve special attention. Dogs showed variable infection rates with this bacterium, with prevalence reaching up to 75% [41]. As for cats, although the studies consulted reported absence of infection or low prevalence [37,42], their tendency to prey on rodents [101] means they should be closely monitored, even more so considering the growing presence of this species, increasingly common in human settlements in the region.

Toxoplasmosis is a disease obligatorily linked to the presence of felids, as only they can act as the final hosts of the protozoan *Toxoplasma gondii* [102]. The great diversity of wild species capable of fulfilling this role in our study area apparently relegates domestic cats to a secondary position compared to other geographical regions [103,104]. Mentions of domestic cat populations vary across the studies consulted, with cats generally considered scarce, and the primary sources of infection identified as the consumption of water contaminated with oocysts or tissue cysts in undercooked meat from intermediate hosts [18,104,105]. However, when domestic cats are abundant, which may occur locally, other infection routes have been reported, such as ingestion or even inhalation of oocysts present in the soil of inhabited areas [106]. The presence of highly virulent strains at the domestic–wildlife interface appears to be responsible for outbreaks affecting multiple people, including some fatal cases [18]. Apart from toxoplasmosis, other intestinal protozoa are scarcely reported in the studies reviewed. There is only a single mention of *Cryptosporidium*, *Giardia*, and *Endolimax nana* on the periphery of the region, already outside the study area [49].

Before our selected timeframe, some studies of canine dirofilariasis caused by *Dirofilaria immitis* were conducted in the Brazilian [107] and Colombian Amazon [108]. Dogs are considered the main source of human filariasis infection, of particular concern among native communities such as the Brazilian Yanomami [109]. Several studies have detected dirofilariosis in dogs [36,47,54,55,56], but only one in cats [42]. Regarding other related species, only one paper mentions *Acanthocheilonema reconditum* [47].

Ectoparasites are represented in this review by mites (genus *Sarcoptes*), fleas (genus *Tunga*), and ticks. Although dogs are the species most often reported as the source of zoonotic infection in humans by the mite *Sarcoptes scabiei*, the causative agent of sarcoptic mange [110], we found only one study referencing this disease [42], suggesting it is likely underreported. It is also a significant disease for wildlife, capable of causing episodes of high morbidity and mortality in susceptible and naïve populations [111,112,113], and has been diagnosed in wild canids from the region, such as the maned wolf (*Chrysocyon brachyurus*) [114,115,116] and crab-eating foxes (*Cerdocyon thous*) [117]. Tungiasis has been widely reported in countries of the region [118,119]; however, it is scarcely documented in our study area [86,87]. Regarding ticks, they are represented by *Rhipicephalus sanguineus* and, particularly, by species of the genus *Amblyomma* (*A. ovale*, *A. scalpturatum*, *A. oblongoguttatum*, *A. latepunctatum*, *A. coelebs*, *A. naponense*) [46]. These arachnids attract considerable interest among researchers, either due to their presence alone or because of the tick-borne diseases associated with them, as conditions such as ehrlichiosis [37,40], rickettsiosis [40,43,45], borreliosis [32], and babesiosis [31,40] are reported. Although one of the studies [36] investigates *Anaplasma phagocytophilum* as *A. platys*, the results are negative. No studies specifically addressing dipteran biting rates were identified. In contrast to other ectoparasites that remain on the host and can be directly collected and quantified, this methodological approach is not applicable to hematophagous flying insects. Given their recognized potential as vectors of multiple pathogens, targeted studies are required to quantify the relative concrete relevance of different dipteran species and their relevance to pathogen transmission dynamics.

Overall, fungi were the least frequently reported pathogens, followed by viruses and bacteria, which is a reasonable outcome in cases where dogs and cats are not common sources of human infection. This is the case of Q fever, caused by *Coxiella burnetii*, for which, although outbreaks originating from pets have been reported [120,121], they are much more frequently associated with contact with ungulates [122]. A similar situation occurs with brucellosis, in which *Brucella canis*, the species most associated with dogs (and which is the agent selected in the present review), is considered much less pathogenic than other members of the genus such as *B. ovis*, *B. melitensis*, or *B. abortus* [123]. However, diagnostic limitations may underestimate its true relevance [124,125].

Contrary to what occurs with trypanosomosis, it has been statistically demonstrated that dog ownership constitutes a protective factor against arboviroses such as Mayaro virus [29,126]. Studies in rural and urban communities of the Brazilian Amazon have found canine prevalence reaching 60.5% [29]. However, cat ownership does not reduce transmission to humans [29] and may even act as a higher transmission risk factor compared to other animals such as poultry or ungulates [126].

*Lyssavirus* (Rabies virus) is the most frequently reported viral agent in the reviewed studies. Its detection occurs both as part of preventive campaigns [33] and in response to human outbreak investigations, where suspected pets involved in aggressive incidents are often euthanized and analyzed [30]. Tracing the origin of outbreaks can be challenging, largely due to the presence of viral reservoirs such as hematophagous bats inhabiting the domestic–wildlife interface [30,31]. Rabies represents one of the zoonoses in which the greatest preventive efforts are invested, including pre-exposure prophylaxis (PEP) campaigns for at-risk personnel, widespread vaccination of the canine population, and surveillance of wild reservoirs.

The recent SARS-CoV-2 pandemic: despite the interest generated and the documented vulnerability of Indigenous populations to infection [127,128], only one article reports contagion in pets from isolated communities [34]. The COVID-19 pandemic exposed the fragility of health systems in Amazonian countries and the particular vulnerability of remote populations, especially Indigenous communities [129,130].

*Sporotrichosis* is considered an emerging disease throughout South America, with numerous studies reporting it [131]. However, we found only one investigation in Manaus city [35], which, with a large clinical sample size, highlights the need for further research given its ease of transmission to humans.

The heterogeneous yet widespread distribution of zoonotic pathogens in domestic animals across Amazonian territories highlights critical shortcomings in environmental governance that directly influence pathogen emergence, persistence, and spillover risk. Strengthening governance frameworks should therefore prioritize a One Health-oriented approach that explicitly integrates animal health surveillance within environmental management and public health policies [132,133]. Given the predominance and high prevalence of parasitic zoonoses, particularly vector-borne and environmentally mediated pathogens, governance strategies should emphasize sustained ecosystem monitoring, vector control, and the mitigation of key environmental drivers, including deforestation, land-use change, and unplanned urbanization, which have been repeatedly linked to altered transmission dynamics [134].

A sampling bias cannot be ruled out either. Peripheral areas and population centers have infrastructures and communication routes that facilitate both the spread of pathogens [135,136] and the work of research teams. However, conducting studies in remote or hard-to-reach areas is much more challenging, where logistical difficulties and cultural differences add further obstacles [137,138]. This may likely lead to undersampling in communities that, although isolated, also keep domestic animals [34]. Most of the studies coincide with areas affected by deforestation (especially in southern and eastern Brazil and northern Bolivia) (see Supplementary Materials Tables S1–S3). Habitat fragmentation and the land-use changes associated with it are considered a major environmental stressor linked to an increased risk of pathogen transmission at the domestic–wildlife interface [9,10,11,12]. Large urban centers represent another point of interest, especially those located in the Amazonian interior, such as Manaus or Iquitos. In these areas, the spread of pathogens is favored by high population density, poor waste management, and the proliferation of vectors [139]. Finally, a minority of results deviate from this trend, occurring in remote areas. Those human communities, although isolated, keep domestic animals that are closely connected to the environment and engage in activities that increase infection risk, such as hunting [43].

The pronounced geographic imbalance in available data further underscores the need to expand surveillance coverage to underrepresented and remote regions of the Amazon Basin. Environmental governance mechanisms should support decentralized and locally implemented monitoring programs, reducing reliance on sporadic, research-driven sampling and enabling continuous data generation. Strengthening institutional coordination among environmental, veterinary, and public health authorities at local, national, and transboundary levels is essential to ensure standardized data collection, interoperability of surveillance systems, and effective information sharing across Amazonian countries [140].

Given the frequent reliance on serological diagnostics and the high seroprevalence reported for multiple pathogens, governance frameworks should promote harmonization of diagnostic protocols and the routine integration of molecular tools to improve detection of active infections and transmission potential. Investment in regional laboratory capacity, workforce training, and long-term surveillance infrastructure is critical to support timely, evidence-based decision-making and outbreak preparedness [134].

Within an integrated strategy, the implementation of legal frameworks that ensure the sovereignty of local and Indigenous communities over their lands, along with the establishment and conservation of strictly protected areas, emerge as key mechanisms to curb deforestation and biodiversity loss [13,141,142], and consequently reduce the risk of emergence of zoonotic pathogens with the potential to cause future epidemic outbreaks. A redistribution of resources should also be promoted, aimed at monitoring neglected tropical pathogens both in areas under human pressure and in isolated communities located in remote regions. Finally, these findings point to an urgent need for inclusive, community-based governance models. The active engagement of Indigenous and rural communities—who often coexist closely with domestic animals and wildlife—is essential for effective environmental stewardship and early detection of zoonotic threats. Policies should support participatory surveillance, community-led reporting systems, and culturally appropriate education initiatives that align biodiversity conservation with health protection. Strengthening environmental governance through inclusive, multisectoral, and regionally coordinated One Health strategies is fundamental to mitigating zoonotic disease risk and enhancing resilience at the human–animal–environment interface in Amazonian ecosystems [132,133,134,143].

## 5. Conclusions

This synthesis of the available evidence indicates that zoonotic pathogens affecting domestic dogs and cats are widely distributed across Amazonian territories, with parasitic infections representing the most frequently reported and epidemiologically dominant group. In contrast, bacterial, viral, and fungal pathogens were reported less frequently and showed more heterogeneous and spatially restricted detection patterns.

The compiled literature reveals a pronounced geographic imbalance in research effort. Most studies were concentrated in Brazil and selected areas of Bolivia, Peru, and Colombia, while extensive portions of the Amazon Basin, particularly Guyana, Suriname, Venezuela, and remote transboundary regions, remain markedly understudied. Research was further clustered in peripheral Amazonian zones and urban centers, areas frequently exposed to anthropogenic stressors such as deforestation and land-use change. This uneven spatial coverage constrains the accurate characterization of pathogen circulation and may mask important zoonotic transmission hotspots.

Methodological limitations further restrict interpretation of the available data. The predominance of cross-sectional designs and heterogeneous diagnostic approaches, coupled with frequent reliance on serological evidence without molecular confirmation, limits the capacity to distinguish prior exposure from active infection and ongoing transmission. These gaps highlight the need for harmonized surveillance protocols integrating complementary diagnostic tools.

Collectively, these findings underscore the urgent need to strengthen inclusive, community-engaged One Health surveillance frameworks that prioritize zoonoses, expand coverage to understudied Amazonian regions, and reinforce monitoring of domestic animals at the human–animal–environment interface. Enhancing cross-sectoral coordination among environmental, veterinary, and public health institutions will be essential to improve early detection, reduce zoonotic risk, and support evidence-based disease prevention strategies across Amazonian ecosystems.

## Figures and Tables

**Table 1:** Zoonotic viral and fungal pathogens.

Pathogen	Location	Prevalence	Diagnostic Technique	Host Species	Reference
**Mayaro virus**	**Brazil**				
	Settlements 150 km northern Manaus	60.5% (52/86)	ELISA-ICC	Dog	[29]
		46.1% (95/206)	ELISA-ICC	Cat	[29]
**Rabies virus**	**French Guiana**				
	Cayenne	0% (0/1)	Not specified	Dog	[30]
		0% (0/5)	Not specified	Cat	[30]
	**Brazil**				
	Cantão State Park (TO)	8% (4/50)	RFFIT	Dog	[31]
		11.1% (1/9)	RFFIT	Cat	[31]
	**Bolivia**				
	Nöel Kempff Mercado NP Boundaries	56% (22/39)	RFFIT	Dog	[32]
	Santa Cruz de la Sierra	50.4% (4694/9308)	FITC–anti-rabies globulin	Dog	[33]
**Severe acute respiratory syndrome coronavirus 2** (SARS-CoV-2)	**Ecuador** Ecuadorian Amazonia (exact location not provided)	66.6% (2/3)	RT-qPCR	Dog	[34]
***Sporothrix* spp.** (*Sporothrix brasilensis*, *S. schenkii*, *S. globosa*)	**Brazil**				
	Several locations in Amazonas Estate (mostly in Manaus)	2798 clinical cases	Culture + qPCR	Cat	[35]

Abbreviations are defined in a dedicated section at the end of the manuscript.

**Table 2:** Zoonotic bacterial pathogens.

Pathogen	Location	Prevalence	Diagnostic Technique	Host Species	Reference
	**Guyana**				
***Anaplasma phagocytophylum/A. platys***	Konashen Community	0% (0/20)	ICT	Dog	[36]
***Borrelia burgdorferi***	**Guyana**				
	Konashen Community	0% (0/20)	ICT	Dog	[36]
	**Bolivia**				
	Nöel Kempff Mercado NP Boundaries	0% (0/22)	IFA	Dog	[32]
***Brucella canis***	**Guyana**				
	Konashen Community	0% (0/20)	ICT	Dog	[36]
	**Bolivia**				
	Nöel Kempff Mercado NP Boundaries	10% (4/40)	SAT, AGID II	Dog	[32]
	**Brazil**				
***Brucella* “smooth”** **(*B. abortus*, *B. melitensis*, *B. suis*, *B. neotomae*)**	Cantão State Park (TO)	0% (0/39)	RBT with Acidified Buffered Antigen	Dog	[37]
***Brucella* spp.**	**Ecuador**				
	Tena, Napo	2.6% (1/39)	ELISA	Dog	[38]
	**Brazil**				
	Cantão State Park (TO)	0% (0/39)	RBT	Dog	[37]
***Coxiella burnetti*** **(Q fever)**	**French Guiana**				
	Cayenne	12.3% (7/57)	CF	Dog	[39]
		0% (0/6)	CF	Cat	[39]
	**Ecuador**				
	Tena, Napo	0% (0/39)	ELISA	Dog	[38]
***Ehrlichia canis*/*E. ewingii***	**Guyana**				
	Konashen Community	10% (2/20)	ICT	Dog	[36]
	**Brazil**				
	Chapadinha (MA)	14.6% (47/322)	IFAT	Dog	[40]
	**Bolivia**				
	Nöel Kempff Mercado NP Boundaries	86% (19/22)	IFA	Dog	[32]
***Leptospira* spp.**	**Ecuador**				
	Nueva Providencia, Orellana	75% (36/48)	mAT + PCR	Dog	[41]
	**Brazil**				
	Cantão State Park (TO)	0% (0/10)	mAT	Cat	[37]
		16.1% (9/56)	mAT	Dog	[37]
	**Bolivia**				
	Nöel Kempff Mercado NP Boundaries	20% (8/40)	mAT	Dog	[32]
***Leptospira borgpetersenii* serovar Hardjo**	**Brazil**				
	Cantão State Park (TO)	16.1% (9/56)	SAM	Dog	[31]
		0% (0/10)	SAM	Cat	[31]
***Leptospira interrogans***	**Bolivia**				
	San Buenaventura (Madidi NP border)	31% (8/26)	mAT	Dog	[42]
		7% (1/14)	mAT	Cat	[42]
***Leptospira interrogans serovars bratislava, canicola, grippotyphosa, hardjo, icterohemorrhagica, pomona***	Konashen Community	0% (0/20)	mAT	Dog	[36]
***Rickettsia amblyommii***	**Brazil**				
	Several locations in Maranhão state	10.2% (160/1560)	IFAT	Dog	[43]
***Rickettsia* spp. (*R. rickettsii*, *R. parkeri*, *R. rhipicephali* & *R. bellii*)**	**Brazil**				
	Chapadinha (MA)	18.9% (61/322)	IFAT	Dog	[40]
		0% (0/322)	PCR	Dog	[40]
	Several locations in Maranhão state	12.6% (196/1560)	IFAT	Dog	[43]
		4.1% (64/1560)	IFAT	Dog	[43]
		4.2% (66/1560)	IFAT	Dog	[43]
	**Bolivia**				
	Nöel Kempff Mercado NP Boundaries	86% (19/22)	IFA	Dog	[32]
	**Peru**				
	Cochabamba	2.3% (1/44)	PCR	Dog	[44]
		68% (30/44)	ELISA	Dog	[44]
**Spotted fever group *Rickettsia* (*R. rickettsii*, *R. parkeri*, and *R. peacockii*)**	**Peru**				
	Iquitos	59.2% (42/71)	ELISA	Dog	[45]
		7.7% (1/13)	ELISA	Cat	[45]
**Typhus group *Rickettsia* (*Rickettsia typhi* and *Reckettsia prowazekii*)**	**Peru**				
	Iquitos	2.8% (2/71)	ELISA	Dog	[45]
		0% (0/13)	ELISA	Cat	[45]

Abbreviations are defined in a dedicated section at the end of the manuscript.

**Table 3:** Zoonotic parasitic pathogens.

Pathogen	Location	Prevalence	Diagnostic Technique	Host Species	Reference
	**Brazil**				
***Amblyomma* spp. (*A. ovale*, *A. scalpturatum*, *A. oblongoguttatum*; *A. latepunctatum*; *A. coelebs*; *A. naponense*)**	Municipal Nat. Park of Porto Velho (RO)	4.9% (9/184)	Stereomicroscopy and dichotomous keys (16S RNA gene sequencing for larvae)	Dog	[46]
	Mapinguari National Park (RO)	22.15% (35/158)		Dog	
		
***Amblyomma tigrinum***	**Peru**				
	Cochabamba	22.7% (10/44)	Taxonomic key + PCR	Dog	[44]
***Acanthocheilonema reconditum***	**Brazil**				
	Marajó (PA)	7.18% (30/418)	PCR and sequencing	Dog	[47]
***Ancylostoma* spp.**	**Brazil**				
	Several locations in SE Acre (AC)	42.1% (72/171)	Coprological flotation	Dog	[48]
	Rolim de Moura (RO)	68.71% (112/163)	Coprology	Dog	[21]
	**Colombia**				
	Cali	11.1% (3/27)	Microscopy + PCR	Dog	[49]
***Babesia canis***	**Brazil**				
	Chapadinha (MA)	16.1% (52/322)	IFAT	Dog	[40]
	Cantão (TO)	10.6% (5/47)	PCR	Dog	[31]
		0% (0/5)	PCR	Cat	[31]
	**Colombia**				
***Cryptosporidium* spp.**	Cali	53.3% (8/15)	Microscopy + PCR	Cat	[49]
		55.6% (14/27)	Microscopy + PCR	Dog	[49]
	**Brazil**				
***Dirofilaria immitis***	Marajó (PA)	2.15% (9/418)	PCR and sequencing	Dog	[47]
	Araguaína (TO)	4.5% (5/111)	TESA-blot	Dog	[50]
	Lábrea (AM)	44.4% (44/99)	PCR	Dog	[51]
	Manaus (AM)	3.7% (28/766)	Blood smear	Dog	[52]
	Ilha do Algodoal (AC)	35.8% (24/67)	Knott’s method + PCR	Dog	[53]
	Porto Velho (RO)	12.8% (93/727)	Immunochromatography	Dog	[54]
	Rio Branco (AC)	Case report (1/1)	Microscopy + ICT + Echocardiography	Dog	[55]
	**French Guiana**				
	Cayenne & Kourou	15.3% (15/98)	HWAT	Dog	[56]
		11.2% (11/98)	qPCR	Dog	[56]
	**Guyana**				
	Konashen Community	10% (2/20)	IFAT	Dog	[36]
	**Bolivia**				
	San Buenaventura (Madidi NP border)	39% (11/28)	ELISA (Antigen)	Dog	[42]
		93% (13/14)	kinetic ELISA	Cat	[42]
	Nöel Kempff Mercado NP Boundaries	33% (13/40)	Occult Heartworm	Dog	[32]
	**Brazil**				
***Dipylidium caninum***	Rolim de Moura (RO)	1.23% (2/163)	Coprology	Dog	[21]
	Several locations in SE Acre (AC)	6.4% (11/171)	Coprological flotation	Dog	[48]
	**Brazil**				
***Echinococcus vogeli***	Southern Acre (AC)	1.54% (1/65)	Coprology (sedimentation) + PCR + sequencing	Dog	[57]
	**Brazil**				
***Echinococcus granulosus***	Southern Acre (AC)	1.54% (1/65)	Coprology (sedimentation) + PCR + sequencing	Dog	[57]
***Endolimax nana***	**Colombia**				
	Cali	13.3% (2/15)	Microscopy + PCR	Cat	[49]
	**Colombia**				
***Giardia* spp.**	Cali	3.7% (1/27	Microscopy + PCR	Dog	[49]
		20% (3/15)	Microscopy + PCR	Cat	[49]
***Leishmania amazonensis***	**Brazil**				
	Belem (PA)	Case report (1/1)	Blood smear + Giemsa Microscopy + PCR-RFLP	Cat	[58]
	Ulianópolis (PA)	45% (101/224)	IFAT	Dog	[59]
		1.8% (4/224)	PCR	Dog	[59]
	**Colombia**				
	Several locations in N and W Colombia	22.2% (10/45)	PCR	Dog	[60]
		26.6% (12/45)	PCR	Dog	[60]
***Leishmania (V.) braziliensis***	**Brazil**				
	Ulianópolis (PA)	40.6% (91/224)	IFAT	Dog	[59]
		30.3% (68/224)	IFAT	Dog	[59]
	Several locations in N and W Colombia	17.7% (8/45)	PCR	Dog	[60]
	Tomé-Açu (PA)	14.2% (3/21)	PCR + sequencing (BLASTn)	Dog	[61]
***Leishmania chagasi***	**Brazil**				
	Araguaína (TO)	54.95% (61/111)	IFAT	Dog	[50]
		51.35% (57/111)	ELISA	Dog	[50]
	**Brazil**				
***Leishmania (V.) guyanensis***	Tomé-Açu (PA)	23.8% (5/21)	PCR + sequencing (BLASTn)	Dog	[61]
	**Colombia**				
***Leishmania panamensis***	Several locations in N and W Colombia	13.3% (6/45)	PCR	Dog	[60]
	**Colombia**				
***Leishmania infantum***	Several locations in N and W Colombia	6.6% (3/45)	PCR	Dog	[60]
	**Guyana**				
	Konashen Community	5% (1/20)	IFAT	Dog	[36]
	**Brazil**				
	Southern Mato Grosso (MT)	Analysis on 46 known positive dogs	MLMT	Dog	[62]
	Labréa (AM)	8% (8/99)	IFAT	Dog	[63]
	Manaus (AM)	39% (60/154)	PCR	Dog	[64]
		20.8% (32/154)	Serology	Dog	[64]
	Marabá (PA)	75.5% (302/400)	Serology with chromatography	Dog	[65]
		59.25% (237/400)	PCR	Dog	[65]
	Urubú Branco, Confresa (MT)	4.4% (5/114)	ELISA	Dog	[66]
	São Luís (MA)	30.4% (32/105)	IFAT	Cat	[67]
		8.5% (9/105)	PCR	Cat	[67]
	Several locations at central Pará (PA)	23.2% (30/129)	ELISA	Dog	[68]
	Tomé-Açu (PA)	57.1% (12/21)	PCR + sequencing (BLASTn)	Dog	[61]
***Leishmania shawi***	**Brazil**				
	Ulianópolis (PA)	43.3% (97/224)	IFAT	Dog	[59]
	**Brazil**				
***Leishmania* spp.**	Corumbá (MS)	50% (31/62)	IFAT + ELISA	Dog	[69]
	Xapuri (AC)	Detected, but not specific prevalence	NNN Culture + direct examination, PCR + sequencing, RFLP and HRM	Dog	[70]
	Xingú river (PA)	15.4% (46/298)	IFAT + ELISA	Dog	[71]
	São Luís (MA)	26.25% (21/80)	IFAT	Cat	[72]
	Tomé-Açu (PA)	83% (30/36)	PCR + sequencing (BLASTn)	Dog	[61]
		4.7% (1/21)			
	**French Guiana**				
	Cayenne & Kourou	(autochthon) 1.7% (3/179), (military working dogs) 5.1% (4/78)	qPCR + sequencing	Dog	[73]
	Cayenne	Clinical case (3 dogs)	ICT + PCR	Dog	[74]
	**Peru**				
	Huánuco Department	26% (251/953)	ELISA + PCR	Dog	[75]
***Rodentolepis (Hymenolepis) nana***	**Brazil**				
	Several locations in SE Acre (AC)	0.58% (1/171)	Coprological flotation	Dog	[48]
	**Brazil**				
***Rhipicephalus sanguineus***	Municipal Nat. Park of Porto Velho (RO)	15.8% (29/184)	stereomicroscopy and dichotomous keys (16S RNA gene sequencing for larvae)	Dog	[46]
	Mapinguari National Park (RO)	16.6% (26/158)		Dog	[46]
	Several locations in Maranhão state (MA)	9.6% (150/1560)	Dichotomous keys	Dog	[43]
	**Brazil**				
***Toxocara* spp.**	Several locations in SE Acre (AC)	18.1% (15/83)	Coprological flotation	Dog	[48]
	**Brazil**				
***Toxoplasma gondii***	Xingú river (PA)	48.8% (124/245)	IFAT	Dog	[71]
	Cantão State Park (TO)	47.8% (22/46)	MAT	Dog	[37]
		80% (8/10)	MAT	Cat	[37]
	Rolim de Moura (RO)	82.2% (376/458)	IFAT	Dog	[76]
	Labréa (AM)	61.6% (61/99)	IFAT	Dog	[63]
	Several locations at central Pará (PA)	69.8% (90/129)	IFAT	Dog	[68]
	Tapirapé comm. (MT)	42.22% (47/114)	IFAT	Dog	[77]
	Karajá comm. (PA)	52.83% (112/212)	IFAT	Dog	[77]
	Manaus (AM)	12.3% (19/154)	IFAT	Dog	[78]
	Rolim de Moura (RO)	82.2% (376/458)	IFAT	Dog	[76]
	Labréa (AM)	61.6% (61/99)	IFAT	Dog	[63]
	**Peru**				
	Nueva Esperanza, Yavari-Mirin basin	94.1% (16/17)	ELISA	Dog	[38]
		100% (4/4)	ELISA	Cat	[38]
	**Colombia**				
	Cali	6.6% (1/15)	Microscopy + PCR	Cat	[49]
	**French Guiana**				
	Cayenne & Kourou	55.7% (49/88)	MAT (>1/20)	Mostly dogs & cats (but also a non-specified number of other species)	[79]
	**Bolivia**				
	San Buenaventura (Madidi NP border)	62% (16/26)	IHA	Dog	[42]
	Nöel Kempff Mercado NP Boundaries	80% (32/40)	IHA	Dog	[32]
***Trichuris vulpis***	**Brazil**				
	Rolim de Moura (RO)	11.66% (19/163)	Coprology	Dog	[21]
	Acre	5.26% (9/171)	Coprological flotation	Dog	[48]
	**Brazil**				
***Trypanosoma cruzi***	Corumbá (MS)	76% (47/62)	IFAT, ELISA and nPCR	Dog	[69]
	**French Guiana**				
	Cayenne & Kourou	5.8% (9/153)	RICT + PCR	Dog	[80]
	**Guyana**				
	Konashen Community	0% (0/20)	IFAT	Dog	[36]
	**Colombia**				
	Talaigua nuevo	18.9 (31/164)	ELISA + IFAT	Dog	[81]
	Several locations in N and W Colombia	13.3% (6/45)	PCR	Dog	[60]
	**Brazil**				
***Trypanosoma evansi***	Corumbá (MS)	73% (45/62)	IFAT and nPCR	Dog	[69]
	Ariquemes (RO)	Clinical case 1 dog	Blood smears and PCR	Dog	[82]
	**Colombia**				
	Vichada	10.5% (49/465).	ELISA + IFAT	Dog	[83]
***Trypanosoma* spp.**	**Brazil**				
	Abaetetuba (PA)	0% (0/11)	Blood culture	Dog	[84]
	**Peru**				
	Tocache, San Martin	100% (1/1)	Blood smear + PCR	Dog	[85]
***Tunga penetrans***	**Brazil**				
	Nossa Senhora do Livramento (AM)	75.6% (59/78)	Taxonomic key	Dog	[86]
	**Ecuador**				
***Tunga trimamillata***	Guayaquil (imported from Loja)	Clinical case (1/1)	Morphological diagnosis (characteristic nodule) + histopathology	Dog	[87]
	**Bolivia**				
***Sarcoptes scabiei***	San Buenaventura (Madidi NP border)	55% (22/40)	ELISA	Dog	[42]

Abbreviations are defined in a dedicated section at the end of the manuscript.

## Data Availability

The raw files of the literature review are available upon request from the corresponding author.

## Supplementary Materials

The following supporting information can be downloaded at: https://www.mdpi.com/article/10.3390/pathogens15010077/s1; Table S1: Zoonotic viral and fungal pathogens (expanded version); Table S2: Zoonotic bacterial pathogens (expanded version); Table S3: Zoonotic parasitic pathogens (expanded version).

## Abbreviations

The following abbreviations are used in this manuscript:

Brazilian States	
(AC)	Acre State
(AM)	Amazonas State
(MA)	Maranhão State
(MS)	Mato Grosso do Sul State
(MT)	Mato Grosso State
(PA)	Pará State
(RO)	Rondônia State
(TO)	Tocantins State
Diagnostic test abbreviations	
AGID II	Agar Gel Immunodiffusion test (Type II)
BLASTn	Basic Local Alignment Search Tool–nucleotide
CF	Complement Fixation
ELISA	Enzyme-linked immunosorbent assay
ELISA-ICC	Enzyme-linked immunoassay with infected cultured cells as antigenic matrix
FITC	anti-rabies globulin: Fluorescein isothiocyanate-conjugated anti-rabies globulin
HRM	High-Resolution Melting Analysis
HWAT	Heartworm Antigen Test
ICT	Rapid immunochromatographic test
IFA	Immunofluorescence assay
IFAT	Indirect Fluorescent Antibody Test
IHA	Indirect Hemagglutination Test
mAT	Microscopic Agglutination Test
MAT	Modified Agglutination Test
MLMT	Microsatellite Marker Analysis
NNN Culture	Novy-Nicolle-MacNeal medium culture
nPCR	nested Polymerase Chain Reaction
PCR	Polymerase Chain Reaction
PCR-RFLP	Polymerase Chain Reaction–Restriction Fragment Length Polymorphism
qPCR	quantitative Polymerase Chain Reaction
RBT	Rose Bengal Test
RFFIT	Rapid Fluorescent Focus Inhibition Test
RFLP	Restriction Fragment Length Polymorphism
RICT	Rapid Immunochromatographic Test
RT-qPCR	Reverse Transcription quantitative Polymerase Chain Reaction
SAM	Serum Agglutination Microscopy
SAT	Slide agglutination test
TESA-blot	Trypomastigote Excreted-Secreted Antigen blot

## Appendix A

The complete search string with Boolean operators used in this Systematic review was: Zoonosis OR zoonotic OR emerging infectious disease OR EID OR neglected tropical disease OR NTD AND Amazon OR Amazonic OR Amazonas OR Amazonia OR Amazonian AND Brazil OR Peru OR Colombia OR Bolivia OR Venezuela OR Ecuador OR Guyana OR Suriname OR French Guiana AND dog OR canine OR cat OR feline.
