# Supplementary material for: 2000–2025: A Quarter of a Century of Studies on Pet Ownership in the Amazon—Epidemiological Implications for Public Health
Source: Pathogens. 2026 Jan 10;15(1):77. doi: 10.3390/pathogens15010077 (PMC12845363; doi:10.3390/pathogens15010077)
Supplement: Supplementary file 1 [file pathogens-15-00077-s001.zip › pathogens-4046805-supplementary.pdf]

Supplementary Table S1. Zoonotic viral and fungal pathogens (expanded version).

Pathogen	Location	Prevalence	Diagnostic technique	Host species	Source	Language	Environment	Management	Reference
**Mayaro virus**	**Brazil**								
	Settlements 150km northern Manaus	60.5% (52/86)	ELISA-ICC	Dog	SA (Q1)	Eng	Preserved	Non protected	[23]
		46.1% (95/206)	ELISA-ICC	Cat	SA (Q1)	Eng	Preserved	Non protected	[23]
**Rabies virus**	**French Guiana**								
	Cayenne	0% (0/1)	Not specified	Dog	CR (Q1)	Eng	Urbanised	Non protected	[24]
		0% (0/5)	Not specified	Cat	CR (Q1)	Eng	Urbanised	Non protected	[24]
	**Brazil**								
	Cantão State Park (TO)	8% (4/50)	RFFIT	Dog	PhD T.	Port	Preserved	Protected	[25]
		11.1% (1/9)	RFFIT	Cat	PhD T.	Port	Preserved	Protected	[25]
	**Bolivia**								
	Nöel Kempff Mercado NP Boundaries	56% (22/39)	RFFIT	Dog	SA (Q2)	Eng	Deforested	Indigenous territory	[26]
	Santa Cruz de la Sierra	50.4% (4694/9308)	FITC–anti-rabies globulin	Dog	SA (Q1)	Eng	Urbanised	Non protected	[27]
**Severe acute respiratory syndrome coronavirus 2** (SARS-CoV-2)	**Ecuador** Ecuadorian Amazonia (exact location not provided)	66.6% (2/3)	RT-qPCR	Dog	CR (Q1)	Eng	Preserved	Indigenous territory	[28]
***Sporothrix* spp** (*Sporothrix brasilensis, S.schenkii, S.globosa*)	**Brazil** Several locations in Amazonas Estate (mostly in Manaus)	2798 clinical cases	Culture + qPCR	Cat	SA (Q1)	Eng	Urbanised	Non protected	[29]

Abbreviations: Source: CR: Case report. Phd T: Doctoral Thesis. SA: Scientific article. Q#: Journal quartile ranking. Language: Eng: English. Port: Portuguese.

Supplementary Table S2. Zoonotic bacterial pathogens (expanded version).

Pathogen	Location	Prevalence	Diagnostic technique	Host species	Source	Language	Environment	Management	Reference
Anaplasma phagocytophylum / A. platys	**Guyana** Konashen Community	0% (0/20)	ICT	Dog	SA (Q1)	Eng	Preserved	Indigenous territory	[30]
Borrelia burgdorferi	**Guyana** Konashen Community	0% (0/20)	ICT	Dog	SA (Q1)	Eng	Preserved	Indigenous territory	[30]
	**Bolivia** Nöel Kempff Mercado NP Boundaries	0% (0/22)	IFA	Dog	SA (Q2)	Eng	Deforested	Non protected	[26]
Brucella canis	**Guyana** Konashen Community	0% (0/20)	ICT	Dog	SA (Q1)	Eng	Preserved	Indigenous territory	[30]
	**Bolivia** Nöel Kempff Mercado NP Boundaries	10% (4/40)	SAT, AGID II	Dog	SA (Q2)	Eng	Deforested	Non protected	[26]
***Brucella* "smooth" (*B. abortus, B. melitensis, B. suis, B. neotomae*)**	**Brazil** Cantão State Park (TO)	0% (0/39)	RBT with Acidified Buffered Antigen	Dog	SA (Q1)	Eng	Preserved	Protected	[31]
***Brucella* spp**	**Ecuador** Tena, Napo	2.6 % (1/39)	ELISA	Dog	SA (Q1)	Eng	Preserved	Non protected	[32]
	**Brazil** Cantão State Park (TO)	0% (0/39)	RBT	Dog	SA (Q1)	Eng	Preserved	Protected	[31]
***Coxiella burnetti* (Q fever)**	**French Guiana** Cayenne	12.3% (7/57)	CF	Dog	SA (Q1)	Eng	Urbanised	Non protected	[33]

		0% (0/6)	CF	Cat	SA (Q1)	Eng	Urbanised	Non protected	[33]
	**Ecuador**								
	Tena, Napo	0 % (0/39)	ELISA	Dog	SA (Q1)	Eng	Preserved	Non protected	[32]
Ehrlichia canis/ E. ewingii	**Guyana**								
	Konashen Community	10% (2/20)	ICT	Dog	SA (Q1)	Eng	Preserved	Indigenous territory	[30]
	**Brazil**								
	Chapadinha (MA)	14.6% (47/322)	IFAT	Dog	SA (Q2)	Eng	Deforested	Non protected	[34]
	**Bolivia**								
	Nöel Kempff Mercado NP Boundaries	86% (19/22)	IFA	Dog	SA (Q2)	Eng	Deforested	Non protected	[26]
Leptospira **spp**	**Ecuador**								
	Nueva Providencia, Orellana	75% (36/48)	mAT + PCR	Dog	SA (Q1)	Eng	Preserved	Protected + Indigenous t.	[35]
	**Brazil**								
	Cantão State Park (TO)	0% (0/10)	mAT	Cat	SA (Q1)	Eng	Preserved	Protected	[31]
		16.1% (9/56)	mAT	Dog	SA (Q1)	Eng	Preserved	Protected	[31]
	**Bolivia**								
	Nöel Kempff Mercado NP Boundaries	20% (8/40)	mAT	Dog	SA (Q2)	Eng	Deforested	Non protected	[26]
Leptospira borgpetersenii **serovar Hardjo**	**Brazil**								
	Cantão State Park (TO)	16.1% (9/56)	SAM	Dog	PhD T	Port	Preserved	Protected	[25]
		0% (0/10)	SAM	Cat	PhD T	Port	Preserved	Protected	[25]
Leptospira interrogans	**Bolivia**								
	San Buenaventura (Madidi NP border)	31% (8/26)	mAT	Dog	SA (Q1)	Eng	Preserved	Indigenous territory	[36]
		7% (1/14)	mAT	Cat	SA (Q1)	Eng	Preserved	Indigenous territory	[36]
Leptospira interrogans serovars bratislava,	Konashen Community	0% (0/20)	mAT	Dog	SA (Q1)	Eng	Preserved	Indigenous territory	[30]

canicola, grippotyphosa, hardjo, icterohemorrhagica, pomona									
Rickettsia amblyommii	**Brazil**<br>Several locations in Maranhão state	10.2% (160/1560)	IFAT	Dog	PhD T	Port	Deforested	Non protected	[37]
Rickettsia spp. (R. rickettsii, R. parkeri, R. rhipicephali & R. bellii)	**Brazil**<br>Chapadinha (MA)	18.9% (61/322)	IFAT	Dog	SA (Q2)	Eng	Deforested	Non protected	[34]
		0% (0/322)	PCR	Dog	SA (Q2)	Eng	Deforested	Non protected	[34]
	Several locations in Maranhão state	12.6% (196/1560)	IFAT	Dog	PhD T	Port	Deforested	Non protected	[37]
		4.1% (64/1560)	IFAT	Dog	PhD T	Port	Deforested	Non protected	[37]
		4.2% (66/1560)	IFAT	Dog	PhD T	Port	Deforested	Non protected	[37]
	**Bolivia**<br>Nöel Kempff Mercado NP Boundaries	86% (19/22)	IFA	Dog	SA (Q2)	Eng	Deforested	Non protected	[26]
	**Peru**<br>Cochabamba	2.3% (1/44)	PCR	Dog	SA (Q2)	Eng	Preserved	Non protected	[38]
		68% (30/44)	ELISA	Dog	SA (Q2)	Eng	Preserved	Non protected	[38]
**Spotted fever group *Rickettsia* (*R. rickettsii, R. parkeri*, and *R. peacockii)***	**Peru**<br>Iquitos	59.2% (42/71)	ELISA	Dog	SA (Q2)	Eng	Urbanised	Non protected	[39]
		7.7% (1/13)	ELISA	Cat	SA (Q2)	Eng	Urbanised	Non protected	[39]
**Typhus group *Rickettsia* (*Rickettsia typhi* and *Reckettsia prowazekii)***	**Peru**<br>Iquitos	2.8% (2/71)	ELISA	Dog	SA (Q2)	Eng	Urbanised	Non protected	[39]
		0% (0/13)	ELISA	Cat	SA (Q2)	Eng	Urbanised	Non protected	[39]

Supplementary Table S3. Zoonotic parasitic pathogens (expanded version).

Pathogen	Location	Prevalence	Diagnostic technique	Host species	Source	Language	Environment	Management	Reference
***Amblyomma* spp (*A. ovale, A. scalpturatum, A. oblongoguttatum; A. latepunctatum; A. coelebs; A. naponense*)**	**Brazil**								
	Municipal Nat. Park of Porto Velho (RO)	4.9% (9%184)	Stereomicroscopy and dichotomous keys (16S RNA gene sequenciation for larvae)	Dog	SA (Q2)	Eng	Preserved	Protected	[40]
	Mapinguari National Park (RO)	22.15% (35/158)		Dog	SA (Q2)	Eng	Preserved	Protected	[40]
Amblyomma tigrinum	**Peru**								
	Cochabamba	22.7% (10/44)	Taxonomic key + PCR	Dog	SA (Q2)	Eng	Preserved	Non protected	[38]
Acanthocheilonema reconditum	**Brazil**								
	Marajó (PA)	7.18% (30/418)	PCR and sequencing	Dog	SA (Q1)	Eng	Preserved	Protected	[41]
***Ancylostoma* spp**	**Brazil**								
	Several locations in SE Acre (AC)	42.1 % (72/171)	Coprological flotation	Dog	SA (Q2)	Eng	Deforested	Non protected	[42]
	Rolim de Moura (RO)	68.71% (112/163)	Coprology	Dog	SA (Q2)	Eng	Deforested	Non protected	[43]
	**Colombia**								
	Cali	11.1% (3/27)	Microscopy + PCR	Dog	SA (Q1)	Eng	Urbanised	Non protected	[44]
Babesia canis	**Brazil**								
	Chapadinha (MA)	16.1% (52/322)	IFAT	Dog	SA (Q2)	Eng	Deforested	Non protected	[34]
	Cantão (TO)	10.6% (5/47)	PCR	Dog	PhD T	Port	Preserved	Protected	[25]

		0% (0/5)	PCR	Cat	PhD T	Port	Preserved	Protected	[25]
	**Colombia**								
***Cryptosporidium* spp**	Cali	53.3% (8/15)	Microscopy + PCR	Cat	SA (Q2)	Eng	Urbanised	Non protected	[44]
		55.6% (14/27)	Microscopy + PCR	Dog	SA (Q2)	Eng	Urbanised	Non protected	[44]
Dirofilaria immitis	**Brazil**								
	Marajó (PA)	2.15% (9/418)	PCR and sequencing	Dog	SA (Q1)	Eng	Preserved	Protected	[41]
	Araguaína (TO)	4.5% (5/111)	TESA-blot	Dog					[45]
	Lábrea (AM)	44.4% (44/99)	PCR	Dog	SA (Q2)	Eng	Urbanised	Non protected	[46]
	Manaus (AM)	3.7% (28/766)	Blood smear	Dog	SA (Q2)	Eng	Urbanised	Non protected	[47]
	Ilha do Algodoal (AC)	35.8% (24/67)	Knott's method + PCR	Dog	SA (Q3)	Eng	Preserved	Protected	[48]
	Porto Velho (RO)	12.8% (93/727)	Immunochromatography	Dog	SA (Q2)	Eng	Urbanised	Non protected	[49]
	Rio Branco (AC)	Case report (1/1)	Microscopy + ICT + Echocardiography	Dog	CR (not rated)	Port	Deforested	Non protected	[50]
	**French Guiana**								
	Cayenne & Kourou	15.3% (15/98)	HWAT	Dog	SA (Q2)	Eng	Urbanised	Non protected	[51]
		11.2% (11/98)	qPCR	Dog	SA (Q2)	Eng	Urbanised	Non protected	[51]
	**Guyana**								
	Konashen Community	10% (2/20)	IFAT	Dog	SA (Q1)	Eng	Preserved	Indigenous territory	[30]
	**Bolivia**								
	San Buenaventura (Madidi NP border)	39% (11/28)	ELISA (Antigen)	Dog	SA (Q1)	Eng	Preserved	Indigenous territory	[36]
		93% (13/14)	kinetic ELISA	Cat	SA (Q1)	Eng	Preserved	Indigenous territory	[36]

	Nöel Kempff Mercado NP Boundaries	33% (13/40)	Occult Heartworm	Dog	SA (Q2)	Eng	Deforested	Indigenous territory	[26]
Dipylidium caninum	**Brazil**								
	Rolim de Moura (RO)	1.23% (2/163)	Coprology	Dog	SA (Q2)	Eng	Deforested	Non protected	[43]
	Several locations in SE Acre (AC)	6.4% (11/171)	Coprological flotation	Dog	SA (Q2)	Eng	Deforested	Non protected	[42]
Echinococcus vogeli	**Brazil**								
	Southern Acre (AC)	1.54% (1/65)	Coprology (sedimentation) + PCR + sequencing	Dog	SA (Q1)	Eng	Deforested	Non protected	[53]
Echinococcus granulosus	**Brazil**								
	Southern Acre (AC)	1.54% (1/65)	Coprology (sedimentation) + PCR + sequencing	Dog	SA (Q1)	Eng	Deforested	Non protected	[53]
Endolimax nana	**Colombia**								
	Cali	13.3% (2/15)	Microscopy + PCR	Cat	SA (Q2)	Eng	Urbanised	Non protected	[44]
***Giardia* spp**	**Colombia**								
	Cali	3.7 % (1/27	Microscopy + PCR	Dog	SA (Q2)	Eng	Urbanised	Non protected	[44]
		20% (3/15)	Microscopy + PCR	Cat	SA (Q2)	Eng	Urbanised	Non protected	[44]
Leishmania amazonensis	**Brazil**								
	Belem (PA)	Case report (1/1)	Blood smear + Giemsa	Cat	SA (Q2)	Eng	Deforested	Non protected	[54]

			Microscopy + PCR-RFLP						
	Ulianópolis (PA)	45% (101/224)	IFAT	Dog	Master's T	Port	Urbanised	Non protected	[55]
		1.8% (4/224)	PCR	Dog	Master's T	Port	Urbanised	Non protected	[55]
	**Colombia**								
	Several locations in N and W Colombia	22.2% (10/45)	PCR	Dog	SA (Q1)	Eng	Deforested	Non protected	[52]
		26.6% (12/45)	PCR	Dog	SA (Q1)	Eng	Deforested	Non protected	[52]
Leishmania (V.) braziliensis	**Brazil**								
	Ulianópolis (PA)	40.6% (91/224)	IFAT	Dog	Master's T	Port	Urbanised	Non protected	[55]
		30.3% (68/224)	IFAT	Dog	Master's T	Port	Urbanised	Non protected	[55]
	Several locations in N and W Colombia	17.7% (8/45)	PCR	Dog	SA (Q1)	Eng	Deforested	Non protected	[52]
	Tomé-Açu (PA)	14.2% (3/21)	PCR + sequencing (BLASTn)	Dog	SA (Q2)	Eng	Deforested	Non protected	[56]
Leishmania chagasi	**Brazil**								
	Araguaína (TO)	54.95% (61/111)	IFAT	Dog	SA (Q2)	Eng	Urbanised	Non protected	[57]
		51.35% (57/111)	ELISA	Dog	SA (Q2)	Eng	Urbanised	Non protected	[57]
Leishmania (V.) guyanensis	**Brazil**								
	Tomé-Açu (PA)	23.8% (5/21)	PCR + sequencing (BLASTn)	Dog	SA (Q2)	Eng	Deforested	Non protected	[56]
Leishmania panamensis	**Colombia**								
	Several locations in N and W Colombia	13.3% (6/45)	PCR	Dog	SA (Q1)	Eng	Deforested	Non protected	[52]
Leishmania infantum	**Colombia**								
	Several locations in N and W Colombia	6.6% (3/45)	PCR	Dog	SA (Q1)	Eng	Deforested	Non protected	[52]
	**Guyana**								

	Konashen Community	5% (1/20)	IFAT	Dog	SA (Q1)	Eng	Preserved	Indigenous territory	[30]
	**Brazil**								
	Southern Mato Grosso (MT)	Analysis on 46 known positive dogs	MLMT	Dog	SA (Q1)	Eng	Deforested	Non protected	[58]
	Labréa (AM)	8% (8/99)	IFAT	Dog	SA (Q3)	Eng	Urbanised	Non protected	[59]
	Manaus (AM)	39% (60/154)	PCR	Dog	SA (Q1)	Eng	Urbanised	Non protected	[60]
		20.8% (32/154)	Serology	Dog	SA (Q1)	Eng	Urbanised	Non protected	[60]
	Marabá (PA)	75.5% (302/400)	Serology with chromatography	Dog	SA (Q2)	Eng	Urbanised	Non protected	[61]
		59.25% (237/400)	PCR	Dog	SA (Q2)	Eng	Urbanised	Non protected	[61]
	Urubú Branco, Confresa (MT)	4.4% (5/114)	ELISA	Dog	SA (Q2)	Eng	Deforested	Indigenous territory	[50]
	São Luís (MA)	30.4% (32/105)	IFAT	Cat	SA (Q1)	Eng	Urbanised	Non protected	[62]
		8.5% (9/105)	PCR	Cat	SA (Q1)	Eng	Urbanised	Non protected	[62]
	Several locations at central Pará (PA)	23.2% (30/129)	ELISA	Dog	SA (Q1)	Eng	Deforested	Protected	[63]
	Tomé-Açu (PA)	57.1% (12/21)	PCR + sequencing (BLASTn)	Dog	SA (Q2)	Eng	Deforested	Non protected	[56]
Leishmania shawi	**Brazil**								
	Ulianópolis (PA)	43.3% (97/224)	IFAT	Dog	Master's T	Port	Urbanised	Non protected	[55]
Leishmania **spp**	**Brazil**								
	Corumbá (MS)	50% (31/62)	IFAT + ELISA	Dog	SA (Q1)	Eng	Urbanised	Non protected	[64]
	Xapuri (AC)	Detected, but not specific prevalence	NNN Culture + direct examination, PCR + sequencing, RFLP and HRM	Dog	SA (Q1)	Eng	Deforested	Non protected	[65]

	Xingú river (PA)	15.4 % (46/298)	IFAT + ELISA	Dog	SA (Q2)	Eng	Deforested	Non protected	[66]
	São Luís (MA)	26.25% (21/80)	IFAT	Cat	Graduate monograph	Port	Urbanised	Non protected	[67]
	Tomé-Açu (PA)	83% (30/36) 4.7% (1/21)	PCR + sequencing (BLASTn)	Dog	SA (Q2)	Eng	Deforested	Non protected	[56]
	**French Guiana**								
	Cayenne & Kourou	(autochthon) 1.7% (3/179), (military working dogs) 5.1% (4/78)	qPCR + sequencing	Dog	SA (Q1)	Eng	Urbanised	Non protected	[68]
	Cayenne	Clinical case (3 dogs)	ICT + PCR	Dog	CR (Q1)	Eng	Urbanised	Non protected	[69]
	**Peru**								
	Huánuco Department	26% (251/953)	ELISA + PCR	Dog	PhD T	Eng	Urbanised	Non protected	[70]
Rodentolepis (Hymenolepis) nana	**Brazil**								
	Several locations in SE Acre (AC)	0,58 % (1/171)	Coprological flotation	Dog	SA (Q2)	Eng	Deforested	Non protected	[42]
Rhipicephalus sanguineus	**Brazil**								
	Municipal Nat. Park of Porto Velho (RO)	15,8% (29/184)	stereomicroscopy and dichotomous keys (16S RNA gene sequencing for larvae)	Dog	SA (Q2)	Eng	Preserved	Protected	[40]
	Mapinguari National Park (RO)	16,6% (26/158)		Dog	SA (Q2)	Eng	Preserved	Protected	[40]
	Several locations in Maranhão state (MA)	9.6% (150/1560)	Dichotomous keys	Dog	PhD T	Port	Deforested	Non protected	[37]
***Toxocara* spp**	**Brazil**								
	Several locations in SE Acre (AC)	18.1% (15/83)	Coprological flotation	Dog	SA (Q2)	Eng	Deforested	Non protected	[42]

	**Brazil**								
	Xingú river (PA)	48,8% (124/245)	IFAT	Dog	SA (Q2)	Eng	Deforested	Non protected	[66]
	Cantão State Park (TO)	47.8% (22/46)	MAT	Dog	SA (Q1)	Eng	Preserved	Protected	[31]
		80% (8/10)	MAT	Cat	SA (Q1)	Eng	Preserved	Protected	[31]
	Lábrea (AM)	61,6 % (61/99)	IFAT	Dog	SA (Q3)	Eng	Urbanised	Non protected	[59]
	Several locations at central Pará (PA)	69,8 % (90/129)	IFAT	Dog	SA (Q1)	Eng	Deforested	Non protected	[63]
	Tapirapé comm. (MT)	42,22 % (47/114)	IFAT	Dog	SA (Q3)	Eng	Deforested	Indigenous territory	[72]
Toxoplasma gondii	Karajá comm. (PA)	52,83 % (112/212)	IFAT	Dog	SA (Q3)	Eng	Deforested	Indigenous territory	[72]
	Manaus (AM)	12,3 % (19/154)	IFAT	Dog	SA (Q2)	Eng	Urbanised	Non protected	[73]
	Rolim de Moura (RO)	82,2 % (376/458)	IFAT	Dog	SA (Q3)	Eng	Deforested	Non protected	[71]
	Lábrea (AM)	61,6 % (61/99)	IFAT	Dog	SA (Q3)	Eng	Urbanised	Non protected	[59]
	**Peru**								
	Nueva Esperanza, Yavari-Mirin basin	94.1% (16/17)	ELISA	Dog	SA (Q1)	Eng	Preserved	Non protected	[32]
		100% (4/4)	ELISA	Cat	SA (Q1)	Eng	Preserved	Non protected	[32]
	**Colombia**								
	Cali	6.6% (1/15)	Microscopy + PCR	Cat	SA (Q2)	Eng	Urbanised	Non protected	[44]
	**French Guiana**								
	Cayenne & Kourou	55.7% (49/88)	MAT (>1/20)	Mostly Dogs & cats (but also non specified number of other species)	SA (Q1)	Eng	Urbanised	Non protected	[74]

	**Bolivia**								
	San Buenaventura (Madidi NP border)	62% (16/26)	IHA	Dog	SA (Q1)	Eng	Preserved	Indigenous territory	[36]
	Nöel Kempff Mercado NP Boundaries	80% (32/40)	IHA	Dog	SA (Q2)	Eng	Deforested	Indigenous territory	[26]
Trichuris vulpis	**Brazil**								
	Rolim de Moura (RO)	11,66 % (19/163)	Coprology	Dog	SA (Q2)	Eng	Deforested	Non protected	[43]
	Acre	5,26 % (9/171)	Coprological flotation	Dog	SA (Q2)	Eng	Deforested	Non protected	[42]
Trypanosoma cruzi	**Brazil**								
	Corumbá (MS)	76% (47/62)	IFAT, ELISA and nPCR	Dog	SA (Q1)	Eng	Urbanised	Non protected	[64]
	**French Guiana**								
	Cayenne & Kourou	5.8% (9/153)	RICT + PCR	Dog	SA (Q2)	Eng	Urbanised	Non protected	[75]
	**Guyana**								
	Konashen Community	0% (0/20)	IFAT	Dog	SA (Q1)	Eng	Preserved	Indigenous territory	[30]
	**Colombia**								
	Talaigua nuevo	18.9 (31/164)	ELISA + IFAT	Dog	Graduate monograph	Spa	Urbanised	Non protected	[76]
	Several locations in N and W Colombia	13.3% (6/45)	PCR	Dog	SA (Q1)	Eng	Deforested	Non protected	[52]
Trypanosoma evansi	**Brazil**								
	Corumbá (MS)	73% (45/62)	IFAT and nPCR	Dog	SA (Q1)	Eng	Urbanised	Non protected	[64]
	Ariquemes (RO)	Clinical case 1 dog	Blood smears and PCR	Dog	CR (Not rated)	Port	Urbanised	Non protected	[77]
	**Colombia**								
	Vichada	10.5% (49/465).	ELISA + IFAT	Dog	SA (Q1)	Eng	Deforested	Non protected	[78]
***Trypanosoma* spp**	**Brazil**								

	Abaetetuba (PA)	0 % (0/11)	Blood culture	Dog	SA (Q1)	Eng	Deforested	Non protected	[79]
	**Peru**<br>Tocache, San Martin	100 % (1/1)	Blood smear + PCR	Dog	CR (Q2)	Eng	Urbanised	Non Protected	[80]
Tunga penetrans	**Brazil**<br>Nossa Senhora do Livramento (AM)	75.6% (59/78)	Taxonomic key	Dog	CR (not rated)	Port	Preserved	Non protected	[81]
Tunga trimamillata	**Ecuador**<br>Guayaquil (imported from Loja)	Clinical case (1/1)	Morphological diagnosis (characteristic nodule) + histopathology	Dog	SA (Q1)	Eng	Urbanised	Non protected	[82]
Sarcoptes scabiei	**Bolivia**<br>San Buenaventura (Madidi NP border)	55% (22/40)	ELISA	Dog	SA (Q1)	Eng	Preserved	Indigenous territory	[36]
